# The Effects of Upper-Body Exoskeletons on Human Metabolic Cost and Thermal Response during Work Tasks—A Systematic Review

**DOI:** 10.3390/ijerph17207374

**Published:** 2020-10-09

**Authors:** Simona Del Ferraro, Tiziana Falcone, Alberto Ranavolo, Vincenzo Molinaro

**Affiliations:** 1 INAIL—Department of Occupational and Environmental Medicine, Epidemiology and Hygiene—Laboratory of Ergonomics and Physiology, via Fontana Candida 1, 00078 Monte Porzio Catone, Rome, Italy; t.falcone-sg@inail.it (T.F.); a.ranavolo@inail.it (A.R.); v.molinaro@inail.it (V.M.); 2 Unit of Advanced Robotics and Human-Centred Technologies, Campus Bio-Medico University of Rome, 00128 Rome, Italy

**Keywords:** exoskeletons, wearable assistive device, metabolic cost, oxygen consumption, thermal comfort, lifting task, overhead work, occupational health, work-related musculoskeletal disorders

## Abstract

Background: New wearable assistive devices (exoskeletons) have been developed for assisting people during work activity or rehabilitation. Although exoskeletons have been introduced into different occupational fields in an attempt to reduce the risk of work-related musculoskeletal disorders, the effectiveness of their use in workplaces still needs to be investigated. This systematic review focused on the effects of upper-body exoskeletons (UBEs) on human metabolic cost and thermophysiological response during upper-body work tasks. Methods: articles published until 22 September 2020 were selected from Scopus, Web of Science, and PubMed for eligibility and the potential risk of bias was assessed. Results: Nine articles resulted in being eligible for the metabolic aspects, and none for the thermal analysis. All the studies were based on comparisons between conditions with and without exoskeletons and considered a total of 94 participants (mainly males) performing tasks involving the trunk or overhead work, 7 back-support exoskeletons, and 1 upper-limb support exoskeleton. Eight studies found a significant reduction in the mean values of the metabolic or cardiorespiratory parameters considered and one found no differences. Conclusions: The reduction found represents a preliminary finding that needs to be confirmed in a wider range of conditions, especially in workplaces, where work tasks show different characteristics and durations compared to those simulated in the laboratory. Future developments should investigate the dependence of metabolic cost on specific UBE design approaches during tasks involving the trunk and the possible statistical correlation between the metabolic cost and the surface ElectroMyoGraphy (sEMG) parameters. Finally, it could be interesting to investigate the effect of exoskeletons on the human thermophysiological response.

## 1. Introduction

Recently, new wearable assistive devices, also called exoskeletons, have been developed with the aim of assisting people during their work activity or rehabilitation.

The American Society for Testing and Materials (ASTM) standard F3323 (ASTM F3323−19 [1]) defines an exoskeleton as a “wearable device that augments, enables, assists, and/or enhances physical activity through mechanical interaction with the body” [1]—i.e., it enhances the power and ability of a person during walking, bending, and lifting [2].

They can be classified as active, passive, and semi-active exoskeletons [3]. Active exoskeletons use actuators (electrical motors, hydraulic, or pneumatics-driven [4]) to support human movement, providing additional strength and increasing the performance of the user. Passive exoskeletons harness the storage energy of springs, dampers, or other materials harvested from human movement to support postures and motions [2,3,5]. Semi-active exoskeletons are a combination of passive and active actuators [3], but they will not be discussed in this review.

Exoskeletons can be distinguished also by considering the supported body parts. From this point of view, they can be classified as lower [6,7], upper, and full-body exoskeletons when they provide support respectively to the lower limbs, upper extremities, and trunk, and both upper and lower extremities [5]. Additionally, in the literature some single-joint exoskeletons are shown [8], but they will not be discussed in this review.

Exoskeletons can be applied with different purposes: in rehabilitation medicine to restore abilities, in the military field to augment the mobility of war-fighters, and in the occupational field where they can play an important role in reducing work-related musculoskeletal disorders (WMDs) caused by manually handling materials or maintaining fixed and awkward postures. These work tasks can cause compression and shear forces at the L5-S1 spine joint, exceeding the tissue tolerance and causing increased trunk muscle co-activations [9,10,11,12] and muscle fatigue [13,14]. The prevalence of WMDs is 42%, of which 22% have symptoms in the lower back and dorsal region, 17% in the wrist and hand, 16% in the neck, and 20% in the shoulder and elbow [15], confirming that work-related low-back disorders (WLBDs) and upper-limb work-related musculoskeletal disorders (ULWMSDs) are the most common. Moreover, according to the Bureau of Labour Statistics, in 2015 WMDs resulting from overexertion in lifting accounted for 31% of the total cases for all workers [16] and of the total WMD cases 80% occurred in private industry workers. In Europe, WMDs remain the most common work-related health problem. Of all workers with this problem, 60% identify WMDs as their most serious issue [17]. In particular, according to the Global Burden of Disease, the proportion of the population who are exposed to ergonomic risk factors for low back pain at work or through their occupation is 81.9% [18]. For all the above-mentioned reasons, exoskeletons used in the occupational field are currently designed to support the trunk and upper limbs.

Despite the fact that today exoskeletons are introduced in different occupational fields—such as the industrial sector [3,5], construction [19,20,21], automotive industry [22], logistics [23], and steel manufacturing [24]—with the promise of improving the quality of work by combining human intelligence with exoskeleton assistance, their use in workplaces still needs to be investigated carefully. Considerations on the suitability of exoskeletons, their costs, and their impacts on the occupational safety and health of workers are required [25]. For this reason, the benefits and risks need to be elucidated in order to investigate their effectiveness [2,25], while also trying to involve a large number of participants in the studies. The European Agency for Safety and Health at Work individualizes some potential risks of injuries due to malfunctioning or during a slip, trip, or fall incident or in terms of the limitation of the user’s overall mobility and possible collisions between exoskeletons and work equipment [25]. Howard et al. [3] add that some exoskeletons can cause the shifting of the user’s centre of gravity, compromising their balance and diminishing their recovery strategy. 

Moreover, the workers’ acceptance is fundamental to allow a widespread use of exoskeletons in workplaces. This acceptance is strongly related to the discomfort and usability of exoskeletons, especially when they are used for a long time. Workers’ acceptance can be tested by performing subjective measurements aiming to investigate aspects related to the physical demand, constraints, perceived usefulness, ease of use, intention to use and performance [26]. There are some issues that remain open regarding the long-term effects of exoskeletons on human biomechanics and physiology [25], the “transference of risk” [2]—i.e., the occurrence of other risks caused by the presence of exoskeletons—and the lack of scientific evidence on the ergonomic aspects related to the use of exoskeletons in workplaces [25].

From an ergonomic point of view, the effectiveness of exoskeletons in workplaces should be investigated by addressing biomechanical, physiological, and thermal aspects.

One of the main reasons for the introduction of exoskeletons in the industrial sector is the attempt to reduce musculoskeletal loads, not eliminated by the engineering process, in order to limit exposure to biomechanical risks. At the same time, it may be important to investigate whether the redistribution of weight can lead to higher amounts of physical stress on other parts of the body when the forces are not transferred to the ground, or to the over-soliciting of limbs when efforts are transferred to the ground through them [3].

With regard to the physiological aspects, among others, the issue is related to the metabolic rate (M), i.e. energy efficiency of workers while using an exoskeleton [27,28]. M represents the total energy developed by oxidation processes that transform the chemical energy contained in food into thermal energy (or total metabolic heat production, H) and mechanical energy used for external work (or total work performed by the body, W) [29] according to the following equation:

(1) H=M−W.

*M* is indispensable in performing both vital functions and daily life and work activities; *H* helps to keep the body temperature relatively constant at around 37 °C.

Generally, most of M is transformed into H. During dynamic work that involves large groups of muscles, about 70–75% of M is converted into H [30], and the mechanical efficiency η ($\eta =\frac{W}{M}$) reaches a maximum of about 30% according to [30], or 20−25% according to [29]. In some industrial activities, η is negligible (η = 0).

M measures the energetic cost of muscular load and gives a numerical index of activity [31]. Since M is difficult to measure directly, it is measured indirectly. As muscles can work for a short time without being provided with oxygen, this represents the major energy source, implying that the indirect measure of M is obtained as a function of the oxygen consumption rate $\dot{V}O_{2}$ ($M=f\left(\dot{V}O_{2}\right)$) by means of the energetic equivalent (EE). EE allows the conversion of the respiratory data into energetic data, as it represents the amount of energy metabolized when 1 l of oxygen is used.

Some methods to determine M are described by the International Organization for Standardization 8896 (ISO 8996:2004) [31]; the method based on the $\dot{V}O_{2}$ measure is considered to be one of the most accurate.

Exoskeletons were designed with the aim to provide external joint moments in order to reduce internal ones and then the muscular forces necessary for the generation of movement. The metabolic cost of generating muscular force over time appears to determine the metabolic cost of movement. The metabolic cost of generating muscular force is determined by the shortening of the muscle motor units that are active during movement. For this reason, it could be useful to understand how exoskeletons positively impact on the energy demand determining a reduction in metabolic cost. On the other hand, the use of an exoskeleton implies that an external structure is put on the worker’s body. In some cases, this structure has a weight that can have an impact on the energy demands during the performed activity, leading, in this way, to an increase in the $\dot{V}O_{2}$. Evaluations of M through the global measurement of $\dot{V}O_{2}$—i.e., related to the whole body—help to understand if the ergonomic intervention (i.e., the use of an exoskeleton) is effective from an energetic point of view.

In the literature, this issue is controversial and not solved because the diversity of exoskeletons and the variety of work tasks make it difficult to formulate a general statement [25].

Finally, thermal aspects seem not to have been discussed yet in the literature. In general, thermal issues represent a part of ergonomic analysis regarding the study of environmental thermal effects on the human thermophysiological response. The increase or decrease in the core temperature could have consequences on the health, performance and comfort of the subject. During work or daily life activities, the human body is in continuous heat exchange with the thermal environment, trying to keep its core temperature around 37 °C [29]. The muscular activity produces internal heat that can be dissipated, if necessary, by the activation of thermoregulation mechanisms (for example, peripheral vasodilatation and sweating), which can vary the skin temperature of the body. Issues related to exoskeletons may be addressed by investigating any possible thermal load added by the assistive device (for example, in the case of working activities performed in hot or cold environments) or any possible thermal discomfort caused by an exoskeleton. For example, an exosuit adds a layer on the skin that could alter heat exchanges with the environment and cause a different thermophysiological response in the user.

In view of the use of exoskeletons in the occupational field for lifting heavy loads or overhead work and in the light of the above statements, there are some questions to be asked:

1. With regard to metabolic aspects, do exoskeletons add physiological strain? Do they increase the metabolic cost of workers during tasks involving the trunk or overhead work?
2. With regard to thermal aspects, has the thermal impact already been investigated? If so, do exoskeletons have an impact on the worker’s thermophysiological response? Furthermore, can exoskeletons guarantee thermal comfort for the workers during their activities?

A systematic review of the evidence on the above-formulated questions would allow to identify, select and critically evaluate relevant primary research and analyze information regarding the subjects recruited in existing studies, the work tasks investigated, the type of exoskeletons tested, the experimental setups used, the metabolic and thermal parameters considered and other biomechanical and physiological results, such as kinematic, kinetic, and surface ElectroMyoGraphy (sEMG) ones. A systematic review of the literature about this crucial and specific current topic of “Industry 4.0” would allow to shed light on the current knowledge of the effects of upper-body exoskeletons on human metabolic cost and thermal response during the execution of work tasks. Finally, it would also identify any challenges for future studies in order to evaluate not-investigated aspects. 

This systematic review has the aim, through a literature search, to:

1. Describe the state-of-the-art recent studies that have investigated the metabolic cost of upper-body exoskeletons (UBEs) for tasks involving the trunk and overhead work during their use in order to understand if they add a metabolic load;
2. Understand if the possible thermal impact of exoskeletons on workers and the aspects related to their thermal comfort have been investigated before.

To do this, we selected from the literature search the studies that respect the eligibility criteria. We provided:

1. A descriptive analysis of their characteristics (Section 3.1.2);
2. An assessment of the potential risk of bias for the selected studies included in this review (Section 3.1.3);
3. The findings of each selected study, distinguishing the back-support exoskeletons (BSEs) during tasks involving the trunk from the upper-limb support exoskeletons (ULSEs) during overhead tasks (Section 3.1.4).

## 2. Materials and Methods 

This study was performed using the systematic review method proposed by the Preferred Reporting Items for Systematic Reviews and Meta-Analysis (PRISMA) [32].

### 2.1. Literature Search Strategy

This systematic review considered articles published until 22 September 2020 and the literature search was performed in a systematic manner with the following selected databases: Scopus, Web of Science and PubMed.

There were two issues of interest in this systematic review [33], one regarding metabolic aspects and one regarding thermal aspects. For each issue, two groups of keywords were identified, one related to the exoskeleton type and one related to the issue. Within the same issue, two groups of keywords were combined in the literature search. The groups of keywords identified for the two issues were:

1. For metabolic aspects, 1st group: “exoskeleton”, ”wearable assistive device”, “passive exoskeleton”, “active exoskeleton”, “trunk exoskeleton”, “upper extremity exoskeleton”, “upper limb exoskeleton”, “exosuit”, “lifting”, “overhead work”;
2. For metabolic aspects, 2nd group: “oxygen consumption”, “oxygen volume”, “metabolic cost”, “metabolic energy”, “metabolism”;
3. For thermal aspects, 1st group: “exoskeleton”, ”wearable assistive device”, “passive exoskeleton”, “active exoskeleton”, “trunk exoskeleton”, “upper extremity exoskeleton”, “upper limb exoskeleton”, “exosuit”;
4. For thermal aspects, 2nd group: “thermal comfort”, “comfort”, “human body temperature”, “thermophysiological response”, “thermal stress”, “stress”, “discomfort”, “local temperature”.

The performed literature search strategy asked each database to carry out a search by keywords, titles and abstracts.

### 2.2. Screening Criteria

The literature search was performed by T.F. and S.D.F. The articles obtained were imported into EndNote X9 library (Clarivate Analytics, Philadelphia, PA, USA) and duplicates were removed. The search was limited to papers published in peer-reviewed journals, chapters of books and conference proceedings.

A three-step approach was performed for screening the manuscripts: firstly, the titles were examined for relevance; secondly, the abstracts were considered. When the information was considered relevant, the full texts were retrieved.

### 2.3. Eligibility Criteria

For both the metabolic and thermal aspects, studies were considered eligible if they were written in English and they investigated subjects using UBEs during work tasks involving the trunk or overhead work. Excluded were narrative and systematic reviews or meta-analyses, studies on prostheses or orthosis and studies with patients.

Furthermore, the following were also excluded:

1. For metabolic aspects, studies without metabolic investigations—i.e., measurements of oxygen consumption;
2. For thermal aspects, studies without measurements of the core or skin temperatures.

Two reviewers (S.D.F., V.M.) individually evaluated the eligibility for all articles by assessing titles and abstracts. Disagreements among reviewers were resolved by scheduling meetings with the other two authors (T.F., A.R.) in order to discuss and solve them.

The reference lists of all the selected articles were also scanned to identify any other eligible articles.

### 2.4. Data Extraction

From the studies selected as eligible, data were extracted according to the Population Intervention Comparison Outcome (PICO) model [34]. A standardized data extraction form was used and a pilot test of the extraction data was performed on three randomly selected included studies and refined accordingly.

The extracted information included:

1. Characteristics of the subjects involved in the study (number of subjects, gender, age, height, weight);
2. Intervention measurement details (type of exoskeleton, experimental setup for the trail performed with the exoskeleton);
3. Comparison measurement details (experimental setup for the trials performed without the exoskeleton);
4. Outcome data when reported. Other outcome parameters provided in the studies were reported for completeness.

### 2.5. Assessment of Bias

A bias represents a characteristic of a study that can introduce a systematic error in the magnitude or direction findings. The potential risk of bias was assessed independently by two authors (S.D.F., V.M.) according to the Cochrane Handbook for Systematic Reviews of Interventions [35] and by using the tool ROBINS-I [36,37], which was developed for the risk of bias assessment of non-randomized studies of interventions. The two authors assessed the following risks of bias:

1. confounding (D1);
2. selection of participants into study (D2);
3. classification of interventions (D3);
4. deviation from the intended study (D4);
5. missing data (D5);
6. measurement of outcomes (D6);
7. selection of the reported result (D7).

## 3. Results

The two performed literature database searches are reported in this section separately, distinguishing the results obtained for metabolic aspects (3.1) and thermal aspects (3.2).

### 3.1. Metabolic Aspects

#### 3.1.1. Study Selection

**Figure 1:**
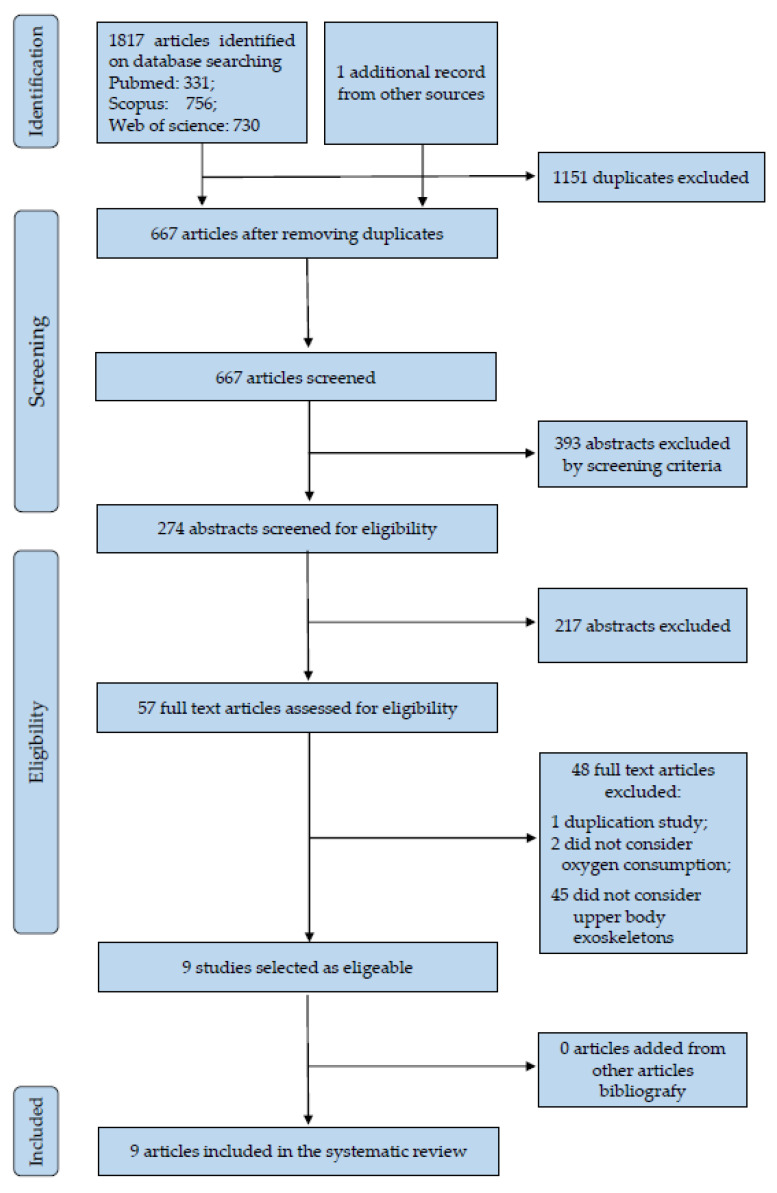
The flowchart of the study selection process.

The study selection process started from the results of the literature database search that yielded 1817 records and a search from other sources (authors’ personal bibliography) that provided one more record, for a total of 1818 records, as shown in Figure 1. After 1151 duplicates were removed, 667 articles were screened based on their title. From this group, 393 abstracts were excluded by the screening criteria and consequently 217 abstracts and 57 full texts were assessed for eligibility. Finally, after having removed 47 articles by the eligibility criteria and 1 duplication study, a total of 9 articles were included in this systematic review.

#### 3.1.2. Characteristics of the Studies

Table 1 shows an overview of the main characteristics of the nine considered studies [26,38,39,40,41,42,43,44,45], following the PICO model and highlighting the type of exoskeleton used. All the articles that met the eligibility criteria are very recent—eight were published between 2019 and 2020 and one, the oldest, in 2014.

A total of 99 subjects were recruited in the included studies, but 94 performed the metabolic test, with a predominance of males (76 males (M), 15 females (F), and 3 subjects whose gender was not specified). The subjects’ mean age varied from less than 25 (six out of nine studies) up to 47 [42].

All the studies were carried out in the laboratory. Most of them (six out of nine, [38,40,41,42,44,45]), chose a repetitive lifting task to be performed in the experimental trial, two out of nine [26,39] selected an overhead working task and one considered a static holding posture of the trunk [43]. All the studies were based on the comparison between the “with exoskeleton condition (WEC)” and “without exoskeleton condition (WOEC)”. One study [38] considered two different exoskeletons; another one [26] tested the same exoskeleton but with different angle-moment settings.

In total, eight different UBEs were investigated: seven BSEs [38,40,41,42,43,44,45] and one ULSE [26,39]. Among them, seven were passive assistive devices and one [45] was an active device.

#### 3.1.3. Risk of Bias

**Figure 2:**
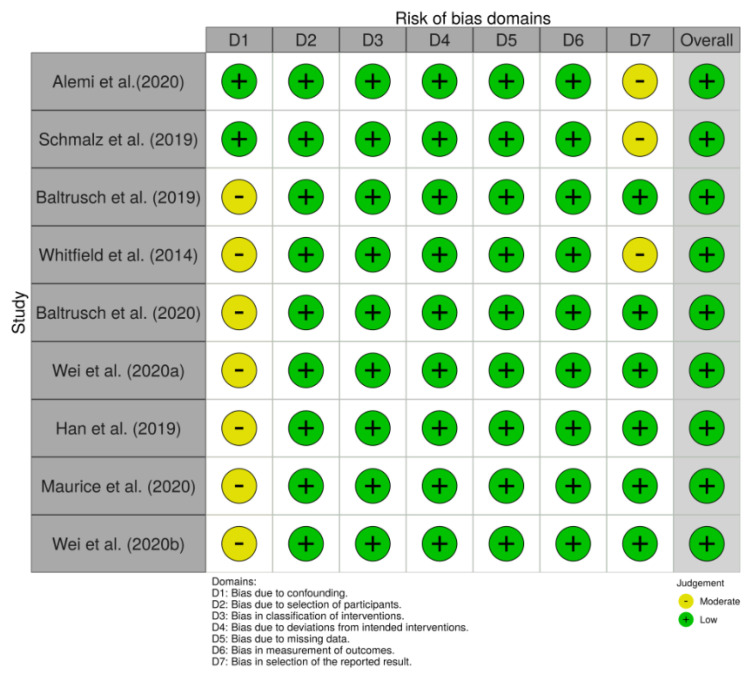
The risk of bias summary: authors’ judgements for each included study and for each considered domain.

**Figure 3:**
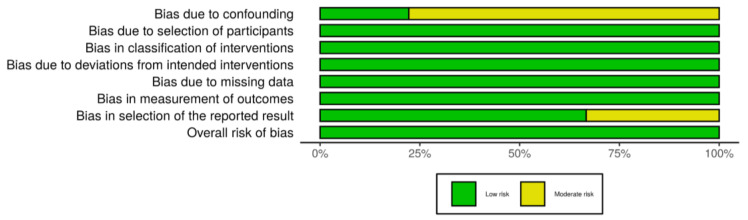
The risk of bias graph: authors’ judgements for each risk of bias reported as percentages across the different studies included in the review.

The results of the risk of bias assessment are reported in the risk of bias summary (Figure 2)—where the authors’ judgements are shown for all the seven considered domains and for each study included in this review, according to [35]—and in the risk of bias graph (Figure 3), where the authors’ judgements are reported for each risk of bias as percentages across the different studies included in this review.

Seven studies [26,40,41,42,43,44,45] had a moderate risk of bias due to confounding, since they recruited only male subjects. Considering that the subjects performing trials in WEC and in WOEC were the same and given the nature of the experimental setup, the authors assigned to all studies a low risk of bias due to the selection of participants, classification of the interventions, deviation from the intended interventions, missing data and measurements of outcomes.

Two studies [40,41] had a moderate risk of bias due to the selection of the reported results, as they did not show the results according to the two samples (male and female) recruited. One study [41] had the same judgement for not having reported among their results all the measurements performed (*HR* data not reported).

#### 3.1.4. The Effect of the Upper-Body Exoskeletons on the Metabolic Cost

##### The Outcome Parameters

Table 2 provides a list and description of all the outcome parameters of interest, while Table 3 shows their values as reported in the included selected studies. One of the eligibility criteria requested that metabolic measurements were performed. All the studies, in fact, carried out $\dot{V}O_{2}$ measurements. Four of them [38,40,42,45] provided results in terms of the energy expenditure rate (EER) or *M*. This implies that they also collected, as they stated, data on the carbon dioxide production rate ($\dot{V}CO_{2}$). The expressions used to convert the respiratory data ($\dot{V}O_{2},\dot{VC}O_{2}$) into energetic data (*M*) are reported in Table 2, when they are explicitly provided in the study. One article [43] provided results in terms of the metabolic cost and another one [45] in terms of the metabolic rate and energy consumption, but they did not provide the calculation performed. Three studies [26,39,41] collected $\dot{V}O_{2}$ and HR data: Schmaltz et al. [39] and Maurice et al. [26] reported both types of data as outcomes, while Whitfield et al. [41] considered only the $\dot{V}O_{2}$ data.

In the following, the findings of the nine included studies are reported, grouped into the studies that investigated BSEs and the studies that investigated ULSEs.

##### The Effects of the BSEs during Tasks Involving the Trunk

Among the seven studies [38,40,41,42,43,44,45] investigating a BSE, six of them found a significant reduction in the average values of the considered parameters in WEC, while one [41] did not find differences between the two conditions tested.

Alemi et al. [38] found a significant reduction in EER in the WEC compared to the WOEC. This reduction appeared overall slightly bigger when using a SuitX than when using a Laevo (respectively, 8% vs. 7.5%). For both the BSEs, bigger percentage changes in energy expenditure (PCEEs) were observed in the symmetric lifting conditions (Laevo, kneeling PCEE = −10.8%, standing PCEE = −8.9%; SuitX, kneeling PCEE = −9.1%, standing PCEE = −12.6%) compared to the asymmetric lifting conditions (Laevo, kneeling PCEE = −5.3%, standing PCEE = −5.5%; SuitX, kneeling PCEE = −6.2%, standing PCEE = −3.7%).

Baltrush et al. [40] tested two settings of a Laevo exoskeleton and two different heights of lifting start (knee height or ankle height). As shown in Table 3, the authors reported for the net metabolic cost ( NMC) the mean values that resulted in being significant ($NMCL_{k,HC}$ = 2.56 W/kg; $NMCL_{a,HC}$ = 4.27 W/kg)—i.e., the mean values obtained for the Laevo HC. Figure 3 reported in the article showed that the NMC values for lifting from ankle height are higher than the values from knee height for both settings of the Laevo. Furthermore, the figure showed that the values of the Laevo LC are comprised between the values obtained in WOEC and those obtained for the Laevo HC, both for lifting from knee height and for ankle height. They also found a significant reduction in *M* for the Laevo HC (17% in LT from knee height and 16% from ankle height), while for the Laevo LC the reduction was lower (7% in LT from knee height and 8% from ankle height) and not significant.

Whitfield et al. [41] tested the effects of the PLAD exoskeleton on the oxygen demand during a highly repetitive lifting task. They found no significant differences in the oxygen consumption between the WOEC and WEC.

The SPEXOR exoskeleton was tested by Baltrush et al. [42]. They found that the use of SPEXOR produced a significant reduction in the NMC mean value of about 18% with respect to the WOEC.

Wei et al. [43] reported the time courses of the median metabolic cost during a static holding posture in WEC and WOEC. The curve obtained in WEC was significantly lower than the curve obtained in WOEC. The authors stated that, in another experiment, they quoted the reduction in metabolic cost to be about 22%, but they did not cite the study.

Han et al. [44] recruited three subjects and used a cardiopulmonary exercise tester to carry out cardio-respiratory measurements, but they exhibited only data referring to the RR and HR. They found a reduction in the average RR and a significant reduction in the average HR (they did not quote these values but, according to the reported data, they could be respectively around 36% and 18%) from before wearing the exoskeleton and after wearing the exoskeleton.

Wei et al. [45] tested the effect of the active exoskeleton MeBot-EXO on the metabolic rate by recruiting three subjects who carried an 8 kg weight from the ground back and forward to a platform at a height of 0.5 m. The results showed that the curves of the metabolic rate and of the energy consumption in WEC are lower that the corresponding curves in WOEC and that the use of the exoskeleton decreased the total energy consumption of the subject by 18%.

##### The Effects of the ULSEs during Overhead Tasks.

A reduction in the average values of the considered parameters in the WEC was also found for the ULSE.

Schmalz et al. [39] found a significant reduction in the $\dot{V}O_{2}$ and HR due to the use of the exoskeleton compared to the WOEC ($\dot{V}O_{2}$: 11% for T1 and 12 % for T2; HR: 6% for T1 and 5% for T2, as reported in the study).

Maurice et al. [26] reported results in terms of box plots, displaying the evolution across blocks of $\dot{V}O_{2}$ and HR for all the participants for WOEC and WEC and confirming a significant reduction in the values (respectively, 33% and 19% on average) in WEC.

### 3.2. Thermal Aspects

#### Study Selection

For the thermal aspects, the results of the literature database search yielded 1284 records. After 621 duplicates were removed, 663 articles were screened on title. From this group, 359 abstracts were excluded by the screening criteria and from then remained 304 records; none met the eligibility criteria—i.e., no article was found to be included in this systematic review.

## 4. Discussion

In an attempt to reduce the risk of WRMDs, new collaborative technologies (cobots and wearbots) are being designed and implemented [49]. Among wearbots, exoskeletons are wearable devices with the aim of assisting humans during their motor daily life activities.

In this systematic review, authors focused on the UBEs that support the back during tasks involving the trunk (BSEs) or the upper limbs during overhead tasks (ULSEs). In particular, it intends to address the effects of BSE and ULSE on the metabolic cost and the human thermophysiological response, aiming to understand if researchers have investigated these two aspects and if these types of exoskeletons really reduce the metabolic cost of workers during their occupational activities.

Generally, many studies [50,51,52,53,54,55,56,57,58,59,60,61,62,63,64,65,66,67,68] investigated the biomechanical aspects, performing measurements of kinematics and electromyography. The study selection process revealed nine articles that, besides the biomechanical analysis, also carried out a metabolic investigation, adding, in this way, some important information about the energy involved in the use of UBEs.

The characteristics of the experimental procedures of these studies suggested some interesting considerations. The samples involved in the studies were constituted mainly by males (76 males vs. 15 females), suggesting that findings related to the metabolic cost cannot be generalized to females, and for this they were assessed as having a moderate risk of bias due to confounding. The results obtained can be useful for workplaces where the working population has a prevalence of males (some industrial sectors), even if they still do not allow us to understand the influence of the gender factor—i.e., if *M* is gender-dependent—during the use of UBEs.

Six studies out of nine recruited young subjects (mean age of less than 25). This can represent a good choice from the recruiting point of view, even though it can represent a weakness if the results need to be applied to a working population that, generally, shows a higher mean age, and also in consideration of active aging at work. Furthermore, all the studies were performed in the laboratory. In the attempt to investigate the effectiveness of exoskeletons in workplaces, measurements should be confirmed in a real work environment, where the workers may not be so young, the lifting tasks can last longer, the selected lifting technique can be different and the frequency can vary within the real setting of the workplaces.

Despite the small number of selected studies, the high number of different UBEs tested (seven BSEs and one ULSE) represents a positive factor. Most of them (seven out of eight) are passive devices, while one is an active device. Passive devices are based on storing and releasing energy during the movements. Passive exoskeletons are adjustable to individual anthropometry, and are less invasive than active exoskeletons from a biomechanical point of view also because active exoskeletons require a heavy drive structure [45]. Passive exoskeletons are preferred in industrial sectors for supporting the back during repetitive lifting tasks or static trunk bending [38]. From this point of view, metabolic investigations, carried out by the nine studies, can add a preliminary contribution to the evaluation of the metabolic cost associated with the use of exoskeletons, highlighting the reduction found in the use of different exoskeletons (WEC vs. WOEC) that needs to be confirmed in real workplaces and for a wider sample of subjects.

The most investigated tasks in the studies (repetitive lifting and overhead work) represent, other than handling low loads at a high frequency, the more physically demanding motor tasks involving the trunk and upper limbs. Some authors investigated specific characteristics of repetitive lifting tasks. One study [38] showed that, in WEC, the symmetry in the lifting task had a net impact on the PCEE (a higher value than those obtained in the asymmetric lifting, see Table 3) for both tested BSEs. The original posture did not seem to have a clear influence on PCEE that can be generalized. The Laevo appeared to be more efficient in symmetric lifting with a kneeling posture (PCEE = −10.8%) than with a standing posture (PCEE = −8.9%) and in asymmetric lifting it was more efficient with a standing posture (PCEE = −5.5%) than with a kneeling posture (PCEE = −5.3%). The SuitX provided a higher PCEE in symmetric lifting with a standing posture (PCEE = −12.6%) than with a kneeling posture (PCEE = −9.1%), and in asymmetric lifting it provided a higher PCEE with a kneeling posture (PCEE = −6.2%) than with a standing posture (PCEE = −3.7%). In another study [40], the authors investigated the effect of the height from which the lifting task started on the NMC, showing that lifting from ankle height required a higher NMC than lifting from knee height also in WEC, both using the Laevo HC and the Laevo LC. The findings of the same study related to the walking task performed using only the Laevo LC show that wearing the exoskeleton increased the NMC. The authors hypothesized that the exoskeleton represented a resistance against hip flexion. These findings suggest that it could be interesting to investigate a combination of the two tasks in order to understand which effect of BSE (increasing or decreasing M) prevails when carrying a load during walking.

With reference to Table 2, some authors [38,40,43,45] decided to exhibit their results in energetic terms, estimating *M* from the respiratory data. It is interesting to notice that, apart from the two studies that did not mention the equation used [43,45], the other two chose different equations—respectively, Brockway’s equation ($M=f(\dot{V}O_{2}$, $\dot{V}CO_{2}$, N)) and the Garby and Astrup equation $(M=f(\dot{V}O_{2}$, $\dot{V}CO_{2}))$. None of the two studies mentioned or applied ISO 8996:2004, the reference standard for the determination of metabolic rate in the field of the ergonomics of the thermal environment, which, at Level 4, suggests two different and well-defined procedures to collect $\dot{V}O_{2}$ and $\dot{V}CO_{2}$ data according to the intensity of the activity. The estimation of M by the measurement of $\dot{V}O_{2}$ and $\dot{V}CO_{2}$ is widely used and represents a compromise between accuracy and ease of measurement [69], even if wearing the mask can cause discomfort for the subject. Kipp et al. [47] found 10 existing equations for calculating energetic cost that differ from the values of the coefficients of $\dot{V}O_{2}$, $\dot{V}CO_{2}$ and Nitrogen N (if considered). They carried out a comparison of those equations across a wide range of exercise intensities and they found an up to 5.2% difference in the calculated metabolic rate between two widely used equations. Regarding the estimation of M, the issue becomes complex when the activity to be estimated is composed of tasks with different levels of intensity (as can happen in workplaces) and the ${\dot{VO}}_{2}$ does not reach a steady state.

The overview reported in Table 2 shows the heterogeneity of the tasks considered, the different types of BSEs tested and in some cases the difference in the body districts involved. For all these factors, it is difficult to make a synthesis or comparison among the numerical results summarized in Table 3. It is possible, anyway, to extrapolate a positive consideration—i.e., that most of the selected studies [26,38,39,42,43,44,45] found a significant reduction in the mean values of the considered metabolic parameters between the WEC and WOEC due to the use of exoskeletons. One [40] found a significant reduction only for the HC setting of the Laevo exoskeleton. Only one study [41] found no differences, although trials revealed a reduced back muscular activity. It is interesting to read that Alemi et al. [38] and Baltrusch et al. [40] mentioned in their papers the different results found by Whitfield et al. [41]. Baltrusch et al. [40] discussed the issue. They hypothesized that the different designed connections between the Laevo (leg pads on the anterior side of the thighs) and PLAD (elastic latex bands that connect the pelvis part to the lower legs) and a change in the self-selected lifting techniques of participants might have been some of the possible causes of the differences in the findings. Some other information on the effects of exoskeletons on M emerges from the study that considered two BSEs in the experiments [38]. The comparison between the Laevo and the SuitX, which differed in the back-unloading mechanism employed, revealed a small difference in the overall PCEE (the SuitX showed a slightly higher value than the Laevo did). For both, larger values were found in the symmetric vs. asymmetric lifting.

In this discussion, we can also highlight that none of the studies performed a biomechanical risk assessment associated with the work task, although this would have been possible using both qualitative and quantitative approaches. For instance, lifting tasks could be classified using kinematic, kinetic, sEMG-based biomechanical risk assessment tools [9,13,70,71,72,73] and also by considering machine learning algorithms such as artificial neural networks [74,75]. The quantification of the risk associated with lifting tasks would have allowed us to relate the exoskeletons’ effectiveness to the risk levels.

As the last consideration related to the metabolic aspects, it is interesting to observe that among all the studies included in this review, which had the merit of performing both biomechanical and physiological analyses, only one [42] tried to associate these two analyses, although without performing statistical correlations.

Baltrush et al. [42] hypothesized a connection in the discussion, arguing that the reduction in metabolic cost in WEC can be explained by an exoskeleton taking over muscular work generated in the hip and the L5-S1 joint, reducing the back muscle activity while on average the movement strategy remains unchanged (no changes in the individual joint angles nor Center Of Mass (COM) displacement). As the metabolic rate measures the energetic cost of muscular load during the execution of work activities, future studies could perform statistical correlation analyses mainly with sEMG parameters.

Unfortunately, thermal aspects were not considered. The literature search performed did not find any articles that addressed this issue. This is indicative of the fact that this issue is perceived as not relevant to investigate in relation to exoskeletons, but it has a potential that should not be underestimated.

The literature search provided records for the keyword “comfort” but not for “thermal comfort”. The concept of comfort in the field of wearbots is related to the user’s acceptance of the exoskeleton—i.e., if the user feels comfortable during the use of an exoskeleton. The “thermal comfort” can represent the psychophysical state in which the user expresses satisfaction with the thermal environment during the use of an exoskeleton. Future studies should investigate the thermophysiological response [76,77,78] of the worker during the use of an exoskeleton. This aspect may be interesting to investigate for particular exoskeletons such as exosuits, whose textile characteristics may affect the heat exchanges between the worker and the environment.

Finally, this review highlights that the effect of exoskeletons on the metabolic cost and on the thermophysiological response is not still sufficiently investigated, although some studies have started to be present in the scientific literature. Investigating these issues represents a great challenge for researchers in order to widen the knowledge on the impact of exoskeletons on humans. Future studies should confirm that working with an exoskeleton is less energy demand than working without an exoskeleton. They should investigate some important aspects, such as the effect of the UBEs on the metabolic cost of males and females, trying to understand if M is gender-dependent during the use of exoskeletons, the dependence of metabolic cost on specific UBE design approaches during tasks involving the trunk and the statistical correlation between the metabolic cost and the sEMG parameters.

In general, there is a need to investigate the generalizability of the exoskeleton effects among a wider range of conditions. In particular, in the occupational field it is important to explore the effectiveness of ergonomic intervention (i.e., the use of exoskeletons) in workplaces, where the task static postures or lifting tasks can last longer than the tasks simulated in the laboratory and the frequency and self-selected lifting technique can vary.

### Limitation of the Present Systematic Review

The main limits are related to the small number of articles that resulted in being eligible, which was due mainly to the narrow eligibility criteria oriented to the occupational field. The number of articles grows significantly if the effect of exoskeletons on metabolic cost is analyzed in some branches of medicine, especially in neurorehabilitation.

## 5. Conclusions

This systematic review focused on the effects of upper-body exoskeletons on human metabolic cost and thermal response during upper-body work tasks. It intends to highlight that still too few studies have carried out experimental trials to investigate the associated metabolic costs, and no studies have been undertaken to investigate the human thermal response during the use of exoskeletons. The evaluation of the metabolic and cardio-respiratory parameters adds important information about the energy involved in the tasks studied to the biomechanical analysis, which generally represents the first aspect investigated. The nine studies that resulted in being eligible by the selection process tested seven back support exoskeletons (six passive and one active) and one upper-limb support exoskeleton. Eight studies found a significant reduction in metabolic cost during the use of exoskeletons, and one found no difference. The reduction found represents a preliminary positive finding that needs to be confirmed in a wider range of conditions (extending the sample also to females and to subjects with an age nearer to that of a work population, and with tasks that last longer). In an attempt to verify the effectiveness of the use of exoskeletons in the occupational field, experimental investigations should be performed in workplaces where work tasks show different characteristics and durations compared to those simulated in a laboratory. Future developments should investigate the dependence of metabolic cost on specific upper-body exoskeleton design approaches during tasks involving the trunk, as well as the possible statistical correlation between the metabolic cost and the sEMG parameters.

Finally, it could be interesting to start to investigate the effect of exoskeletons on the human thermophysiological response, which represents a complementary aspect in the evaluation of the effectiveness of ergonomic intervention represented by the use of exoskeletons.

## Figures and Tables

**Table 1:** Descriptive analysis of the studies considered in the review according to the PICO method.

Ref.	Population		Intervention (WEC)		Comparison (WOEC)	Outcome		
Author (year)	Subjects Involved in the Study	Task	Exoskeleton Tested	Experimental Setup	Experimental Setup	Metabolic Measured Parameters	Metabolic Results Considered	Other Results Reported
Alemi et al. (2020) [38]	18 young subjects (9 M, 9 F) age: M: 24.4 ± 4.5 y F: 25.1 ± 3.8 y height: M: 176.5 ± 5.5 cm F: 167.4 ± 3.5 cm BM: M: 78.5 ± 7.0 kg F: 67.6 ± 9.4 kg BMI: M: 25.2 ± 2.7 kg/m^2^ F: 24.1 ± 3.4 kg/m^2^	Repetitive lifting	SuitX Laevo V2.5	Trial: cycle of lifting/lowering a 6.8 kg panel for 5 min; f: 5 lifting/lowering cycles per min. For each exoskeleton, four conditions were tested which combine: - 2 LS: symmetric and asymmetric lifting; - 2 posture: standing and kneeling. 8 trials per subject.	Trial: cycle of lifting/lowering a panel for 5 min; f: 5 lifting/lowering cycles per min. Four conditions were tested which combine: - 2 LS: symmetric and asymmetric lifting - 2 posture: standing and kneeling. 4 trials per subject.	$\dot{\dot{V}O_{2}}$ $\dot{\dot{V}CO_{2}}$	EER, PCEE	sEMG: peak activity of normalized signals from trunk, lower, and upper limb muscles; perceived discomfort and balance; overall usability.
Schmalz et al. (2019) [39]	12 healthy subjects (6 M, 6 F) age: 24 ± 3 y height: 176 ± 15 cm weight: 73 ± 15 kg Subjects were divided in 2 groups (G1, G2)	Overhead working	PAEXO	Sequence of sessions G1: WE+B+WOE+B+WE. Sequence of sessions G2: WOE+B+WE+B+WOE. Each session WE was a sequence of 2 tasks (T1, T2) of 5 min with a break of 10 min. T1: screwing nuts continuously; T2: drilling using an electric drill. The order of T1 and T2 was chosen randomly. B is of 30 min. G1 performs 2 WE sessions. G2 performs 1 WE session.	Each WOE is a sequence of 2 tasks (T1, T2) of 5 min with a break of 10 min. T1: screwing nuts continuously; T2: drilling using an electric drill. G1 performs 1 WOE session. G2 performs 2 WOE session.	$\dot{V}O_{2}$ HR	$\dot{V}O_{2,\mathrm{rest}}$ $\dot{V}O_{2,T1}$ $\dot{V}O_{2,T2}$ $HR_{rest}$ $HR_{T1}$ $HR_{T2}$	Kinematics: shoulder and elbow angles; sEMG: amplitude and Muscle Fatigue Index from trunk and upper limb muscles.
Baltrusch et al. (2019) [40]	13 M subjects for WT 11 M subjects for LT age: 28.9 ± 4.4 y height: 180 ± 4 cm weight: 76.9 ± 12.0 kg	Walking, Repetitive lifting	Laevo HC Laevo LC	WT: 5 min of walking on treadmill with Laevo LC at PWS and PWSX. 2 trials for each subject. LT: cycle of lifting/lowering a 10-kg box; f: 6 lifting/lowering cycles per min. LT was performed for Laevo HC and LC and from 2 heights: ankle and knee height. 4 trials for each subject. 30 s of rest between each trial.	WT: 5 min of walking on treadmill at PWS and PWSX. 2 trials for each subject. LT: cycle of lifting/lowering a 10-kg box; f: 6 lifting/lowering cycles per min. LT was performed from 2 heights: ankle and knee height. 2 trials for each subject. 30 s of rest between each trial.	$\dot{\dot{V}O_{2}}$ $\dot{\dot{V}CO_{2}}$	NMCL NMCW	Kinematics: knee, hip, lumbar flexio-extension and trunk inclination ROM; ROM of the body COM. sEMG: normalized signals from trunk and lower limb muscles.
Whitfield et al. (2014) [41]	15 M subjects age: 22.1 ± 2.6 y height: 1.81 ± 0.08 m weight: 81.6±9.2 kg	Repetitive lifting	PLAD	LT: cycle of lifting/lowering a box (average weight 8.9 ± 1.6kg) for 15 min; f: 6 lifting/lowering cycles per min.	LT: cycle of lifting/lowering a 8.9-kg box for 15 min; f: 6 lifting/lowering cycles per min.	$\dot{VO_{2}}$ HR	$\dot{V}O_{2,\mathrm{avg}}$	Kinematics: maximum knee flexion angles. sEMG: normalized sEMG signals from lower limb muscles.
Baltrusch et al. (2020) [42]	11 M employees of KLM age: 47.4 ± 7.1 y height: 175 ± 7.0 cm weight: 84.6 ± 15 kg (finally 10 M were considered because one failed). Enrolled subjects with and without a history of LBP	Symmetric repetitive lifting	SPEXOR	LT: cycle of lifting/lowering a 10-kg box from ankle height to hip height for 5 min; f: 8 lifting/lowering cycles per min.	LT: cycle of lifting/lowering a 10-kg box from ankle height to hip height for 5 min; f: 8 lifting/lowering cycles per min.	$\dot{\dot{V}O_{2}}$ $\dot{\dot{V}CO_{2}}$	NMC	Kinematics: knee, hip, trunk and L5-S1 flexion-extension angles; Range of motion of COM; Mechanical work generated at knee, hip, trunk and L5-S1 joint. sEMG: normalized signal from trunk muscles.
Wei et al. (2020a) [43]	8 M subjects age: 24.4 ± 2.54 y height: 172.1 ± 5.89 cm weight: 65.25 ± 6.98 kg	Static holding posture	MeBot-EXO (passive device)	SST: forward trunk flexion at an angle of 50–55 degrees and this stooping position is held for 5 min. Each SST was repeated three times.	SST: forward trunk flexion at an angle of 50–55 degrees and this stooping position is held for 5 min. Each SST was repeated three times.	$\dot{V}O_{2}$	MMC	sEMG: RMS of normalized signals from trunk muscles.
Han et al. (2019) [44]	3 subjects age: (^1^) height: (^1^) weight: (^1^)	Repetitive lifting	Passive energy-storing booster exoskeleton	LT: 20 cycles of lifting/lowering a 9-kg object. Three LT for each subject. f not considered.	?	RR HR	RRHR	
Maurice et al. (2020) [26]	12 M subjects age: 23.4 ± 1.2 y height: 179.3 ± 5.9 cm weight: 72.7 ± 5.4 kg	Overhead working	PAEXO	Overhead pointing task: moving the power drill as far as possible from a starting point to a target and remain on the target for 2 s. Session: 5 blocks of 24 pointing movement each with a break of 30 s between blocks.	Overhead pointing task: moving the power drill as far as possible from a starting point to a target and remain on the target for 2 s. Session: 5 blocks of 24 pointing movements, each with a break of 30 s between blocks.	$\dot{V}O_{2}$ HR	${\dot{VO}}_{2,avg}$ NHR	sEMG: RMS of normalized signals from shoulder and trunk muscles. Kinetics: RMS of the CoP displacement and velocity; Duration of movement. Kinematics: temporal profiles and maximum joint angles of the shoulder, elbow, lower back and hip.
Wei et al. (2020b) [45]	7 M for the sEMG measurement. 3 M subjects for the metabolic measurement age: 24 ± 3 y height: 170 ± 5 cm weight: 70 ± 5 kg	Repetitive semi-squat lifting	MeBot-EXO (active device)	LT: carrying an 8 kg weight from the ground back and forward to a platform at a height 0.5 m for 5 min. Three LT for each subject. f not declared.	LT: carrying an 8 kg weight from the ground back and forward to a platform at a height 0.5 m for 5 min. Three LT for each subject. f not declared.	?	M EC	Kinematics: temporal profiles of hip (right and left) and back angles. sEMG: RMS of rectified signals from trunk muscles.

Avg: average; B: break; BM: body mass; BMI: body mass index; COM: center of mass; CoP: center of pressure; EC: energy consumption; EMG: electromyography; EER: energy expenditure rate; F: female; f: frequency; G1: group 1; G2: group 2; HC: high-cam (supporting predominantly at trunk bending angles from 0−20 degrees); HR: heart rate; LBP: low back pain; LC: low-cam: (supporting predominantly at trunk bending angles >20 degrees); LS: level of symmetry; LT: lifting trial; M: male; MFI: mean fatigue index; min: minute; NMC: net metabolic cost; NMCL: net metabolic cost of lifting; NMCW: net metabolic cost of walking; NHR: normalized heart rate; PCEE: percentage changes in energy expenditure; PWS: preferred walking speed without the exoskeleton; PWSX: preferred walking speed with the exoskeleton; RMS: root mean square; ROM: range of motion; RR: respiratory rate; s: second; SST: static stooping bending; T1: task 1; T2: task 2; $\dot{V}CO_{2}$: carbon dioxide production rate; $\dot{V}O_{2}$: oxygen consumption rate; WT: walking test; WE: with exoskeleton; WEC: with exoskeleton condition; WOE: without exoskeleton; WOEC: without exoskeleton condition; y: years; (^1^): data not reported; ?: not specified.

**Table 2:** Description of all the outcome parameters from the eligible studies.

Outcome Measure	Unit	Description and/or Calculation	Reference
EER	Kcal/min*kg	Energy expenditure rate estimated using the Brockway equation (1987) [46], reported as 1 $16.58\cdot \dot{V}O_{2\;}4.51\cdot \dot{V}CO_{2}-5.9\cdot N$ in [47]. Alemi et al. [38] considered the mean value over the last 2 min of each trial, normalized with respect to the mass. Authors highlighted that this approach accounts for both the participant body mass and the mass of a BSE (if used).	Alemi et al. 2020 [38]
PCEE	%	Percentage changes in energy expenditure considering WEC vs. WOEC.	
$\dot{V}O_{2,\mathrm{rest}}$	ml/min*kg	Oxygen consumption rate at rest, mean value over the last minute before starting T1 or T2, normalized with respect to the body mass.	Schmalz et al. 2019 [39]
$\dot{V}O_{2,T1}$	ml/min*kg	Oxygen consumption rate of T1, mean value over the last minute of the task, normalized with respect to the body mass.	
$\dot{V}O_{2,T2}$	ml/min*kg	Oxygen consumption rate of T2, mean value over the last minute of the task, normalized with respect to the body mass.	
$HR_{rest}$	beat/min	Heart rate at rest, mean value over the last minute before starting T1 or T2.	
$HR_{T1}$	beat/min	Heart rate of T1, mean value over the last minute of the task.	
$HR_{T2}$	beat/min	Heart rate of T2, mean value over the last minute of the task.	
$NMCL_{k,LC}$	W/kg	Net metabolic cost was calculated by subtracting the resting metabolic cost from the total metabolic rate during lifting from knee height with Laevo LC. The metabolic cost of lifting was estimated using the Garby and Astrup equation [48] for the energetic expenditure, reported as $EE=16.04\cdot \dot{V}O_{2}+4.94\cdot \dot{V}CO_{2}$ in [47]. Flow rates were averaged over the last 2 min of the trial and were normalized to the body mass.	Baltrush et al. (2019) [40]
$PRMCL_{k,LC}$	%	Percentage of reduction in metabolic cost between WEC and WOEC for lifting from knee height, with the Laevo LC.	
$NMCL_{k,HC}$	W/kg	Net metabolic cost was calculated by subtracting the resting metabolic cost from the total metabolic rate during lifting from knee height with Laevo HC. The metabolic cost of lifting was estimated using the Garby and Astrup equation [48] for the energetic expenditure, reported as $EE=16.04\cdot \dot{V}O_{2}+4.94\cdot \dot{V}CO_{2}$ in [47]. Flow rates were averaged over the last 2 min of the trial and were normalized to the body mass.	
$PRMCL_{k,HC}$	%	Percentage of reduction in metabolic cost between WEC and WOEC for lifting from knee height with the Laevo HC.	
$NMCL_{a,LC}$	W/kg	Net metabolic cost was calculated by subtracting the resting metabolic cost from the total metabolic rate during lifting from ankle height with the Laevo LC. The metabolic cost of lifting was estimated using the Garby and Astrup equation [48] for the energetic expenditure, reported as $EE=16.04\cdot \dot{V}O_{2}+4.94\cdot \dot{V}CO_{2}$ in [47]. Flow rates were averaged over the last 2 min of the trial and were normalized to the body mass.	
$PRMCL_{a,LC}$	%	Percentage of reduction in metabolic cost between WEC and WOEC for lifting from ankle height with the Laevo LC.	
$NMCL_{a,HC}$	W/kg	Net metabolic cost was calculated by subtracting the resting metabolic cost from the total metabolic rate during lifting from ankle height with the Laevo HC. The metabolic cost of lifting was estimated using the Garby and Astrup equation [48] for the energetic expenditure, reported as $EE=16.04\cdot \dot{V}O_{2}+4.94\cdot \dot{V}CO_{2}$ in [47]. Flow rates were averaged over the last 2 min of the trial and were normalized to the body mass.	
$PRMCL_{a,HC}$	%	Percentage of reduction in metabolic cost between WEC and WOEC for lifting from ankle height with the Laevo HC.	
$MCW_{PSW}$	J/m*kg	The metabolic cost of walking with and without Laevo LC at PSW was estimated using the Garby and Astrup equation [48] for the energetic expenditure, reported as $EE=16.04\cdot \dot{V}O_{2}+4.94\cdot \dot{V}CO_{2}$ in [47]. Flow rates were averaged over the last 2 min of the trial. Metabolic cost was normalized to the body mass and the walking speed.	
$MCW_{PSWX}$	J/m*kg	The metabolic cost of walking with and without the Laevo LC at PSWX was estimated using the Garby and Astrup equation [48] for the energetic expenditure, reported as $EE=16.04\cdot \dot{V}O_{2}+4.94\cdot \dot{V}CO_{2}$ in [47]. Flow rates were averaged over the last 2 min of the trial. Metabolic cost was normalized to the body mass and the walking speed.	
${\dot{VO}}_{2,avg}$	ml/kg*min	Average oxygen consumption, normalized by weight.	Whitfield et al. (2014) [41]
HR	Beat/min	Heart rate.	
NMCL	W/kg	Net metabolic cost was calculated by subtracting the resting metabolic cost from the total metabolic rate during lifting. The total metabolic cost of lifting from ankle to hip height with SPEXOR was estimated using the Garby and Astrup equation [48] for the energetic expenditure, reported as $EE=16.04\cdot \dot{V}O_{2}+4.94\cdot \dot{V}CO_{2}$ in [47]. Flow rates were averaged over the last 2 min of the trial and normalized with respect to the body mass.	Baltrush et al. (2020) [42]
PRMCL	%	Percentage of reduction in metabolic cost between WEC and WOEC for lifting.	
MMC	Kcal/min*kg	Median metabolic cost of energy (^1^) during static holding posture normalized by weight.	Wei et al., (2020a) [43]
RR	l/min	Respiratory rate collected before and after wearing the exoskeleton.	Han et al. (2019) [44]
$HR_{avg,bef}$	beat/min	Average heart rate before wearing the exoskeleton.	
$HR_{avg,aft}$	beat/min	Average heart rate after wearing the exoskeleton.	
${\dot{VO}}_{2,norm}$	ml/kg*min	Average oxygen consumption, normalized by weight.	Maurice et al. (2020) [26]
$HR_{norm}$		Average heart rate normalized using the maximum and minimum values of the participant in WOEC.	
M	Kcal/min*Kg	Metabolic rate for 5 min during continuous manual material handling, normalized by weight.	Wei et al. (2020b) [45]
EC	Kcal	Energy consumption for 5 min during continuous manual material handling.	

LC: low-cam (supporting predominantly at trunk bending angles >20 degrees); HC: high-cam (supporting predominantly at trunk bending angles from 0 to 20 degrees); l: liter; min: minute; ml: milliliter; kcal: kilocalories; kg: kilogram; J: joule; PWS: preferred walking speed without the exoskeleton; PWSX: preferred walking speed with the exoskeleton; T1: task 1; T2: task 2; W: watt; (^1^): it is not specified how the metabolic cost was calculated.

**Table 3:** The values of the outcome parameters of interest reported in the selected studies.

			Exoskeleton Condition			Control Condition	
Ref.	Outcome	Subject	Exoskeleton	Task	M (SD)	Task	M (SD)
Alemi et al. (2020) [38]	EER (Kcal/min*Kg)	18 (9M, 9F)	Laevo	All trials	0.07 (0.02) (*)	All trials	0.075 (0.019)
			SuitX		0.068 (0.018) (*)		
	PCEE (%)		Laevo	All trials	−7.5		
			SuitX		−8		
			Laevo	KA	−5.3		
				KS	−10.8		
				SA	−5.5		
				SS	−8.9		
			SuitX	KA	−6.2		
				KS	−9.1		
				SA	−3.7		
				SS	−12.6		
Schmalz et al. (2019) [39]	$\dot{V}O_{2,\mathrm{rest}}$ (ml/min*Kg)	12 (6M,6F)	Paexo	rest	4.1 (^2^)	rest	3.9 (^2^)
	$\dot{V}O_{2,T1}$ (ml/min*Kg)			T1	5.2 (^2^) (*)	T1	5.8 (^2^)
	$\dot{V}O_{2,T2}$ (ml/min*Kg)			T2	6.6 (^2^) (*)	T2	7.4 (^2^)
	$HR_{rest}$ (beat/min)			rest	75 (^2^)	rest	74 (^2^)
	$HR_{T1}$ (beat/min)			T1	98 (^2^) (*)	T1	103 (^2^)
	$HR_{T2}$ (beat/min)			T2	93 (^2^) (*)	T2	99 (^2^)
Baltrush et al. (2019) [40]	$NMCL_{k,LC}$ (W/kg) $PRMCL_{k,LC}$ (%)	11 M	Laevo LC	LT- knee height	7	LT- knee height	3.09 (0.92)
	$NMCL_{k,HC}$ (W/kg) $PRMCL_{k,HC}$ (%)		Laevo HC		2.56 (0.52) (*) 17 (*)		
	$NMCL_{a,LC}$ (W/kg) $PRMCL_{a,LC}$ (%)		Laevo LC	LT- ankle height	8	LT-ankle height	5.06 (1.11)
	$NMCL_{a,HC}$ (W/kg) $PRMCL_{a,HC}$ (%)		Laevo HC		4.27 (0.6) (*) 16 (*)		
	$NMCW_{PSW}$ (J/m*kg)	13 M	Laevo LC	WT-PSW	(^1^) $MCW_{WOEC,PSW}<MCW_{WEC,PSW}$ (increase of 12%)	WT-PSW	(^1^) $MCW_{WOEC,PSW}<MCW_{WEC,PSW}$
	$NMCW_{PSWX}$ (J/m*kg)		Laevo LC	WT-PSWX	(^1^) $MCW_{WOEC,PSWX}<MCW_{WEC,PSWX}$ (increase of 17%)	WT-PSWX	(^1^) $MCW_{WOEC,PSWX}<MCW_{WEC,PSWX}$
Whitfield et al. (2014) [41]	${\dot{VO}}_{2,avg}$ (ml/kg*min) HR (beat/min)	15 M	PLAD	LT	17.7 (2.6) 116.0 (14.8)	LT	17.9 (2.4) 117.7 (17.0)
Baltrush et al. (2019) [42]	NMC (W/kg) PRMCL (%)	10 M	SPEXOR	LT	4.64 (1.38) (*) 18	LT	5.63 (1.26)
Wei et al. (2020a) [43]	MMC (Kcal/min*Kg)	8 M	MeBot-EXO	Static holding posture	(^2^) (*) $MMC_{WEC}<MMC_{WOEC}$	Forward torso flexion	(^2^) $MMC_{WOEC}>MMC_{WEC}$
Han et al. (2019) [44]	RR (1/min)	3 subjects	Passive energy-storing booster exoskeleton	LT	16		25
	$HR_{avg}$ (beat/min)				94 (*) (after using exoskeleton)		114 (*) (before using exoskeleton)
Maurice et al. (2020) [26]	${\dot{VO}}_{2,norm}$ (ml/kg*min)	12 M	PAEXO	Overhead pointing task	(^2^) (*) ${\dot{VO}}_{2,norm,WEC}<{\dot{VO}}_{2,norm,WOEC}$ by 33%	Overhead pointing task	(^2^) (*) ${\dot{VO}}_{2,norm,WOEC}$>${\dot{VO}}_{2,norm,WEC}$
	$HR_{norm}$				(^2^) (*) $HR_{norm,WEC}<HR_{norm,WOEC}$ by 19%		(^2^) (*) $HR_{norm,WOEC}>HR_{norm,WEC}$
Wei et al. (2020b) [45]	M	3 M	MeBot-EXO	LT	(^2^) (*) $M_{WEC}<M_{WOEC}$	**LT**	(^2^) (*) $M_{WOEC}>M_{WEC}$
	EC				17.9 (maximum value at the 5th minute)		22.9 (maximum value at the 5th minute)

LC: low-cam: (supporting predominantly at trunk bending angles >20 degrees); HC: high-cam (supporting predominantly at trunk bending angles from 0 to 20 degrees); EER: energy expenditure rate: F: female; $HR_{avg}$: average heart rate; $HR_{norm}$: average heart rate normalized using the maximum and minimum values of the participant in WOEC; KA: kneeling posture, asymmetric lifting/lowering; KS: kneeling posture, symmetric lifting/lowering; M: male; NW: no exoskeleton; NMC: net metabolic cost; $NMCL_{a,LC}$: net metabolic cost during lifting from ankle with the Laevo LC; $NMCL_{a,HC}$: net metabolic cost during lifting from ankle with the Laevo HC; $NMCL_{k,LC}$: net metabolic cost during lifting from knee with the Laevo LC; $NMCL_{k,HC}$: net metabolic cost during lifting from knee with the Laevo HC; PCEE: percentage changes in energy expenditure; PRMCL: percentage of reduction in metabolic cost between WEC and WOEC for lifting; $PRMCL_{a,LC}$: percentage of reduction in metabolic cost between WEC and WOEC for lifting from ankle height with the Laevo LC; $PRMCL_{a,HC}$: percentage of reduction in metabolic cost between WEC and WOEC for lifting from ankle height with the Laevo HC; $PRMCL_{k,LC}$: percentage of reduction in metabolic cost between WEC and WOEC for lifting from knee height with the Laevo LC; $PRMCL_{k,HC}$: percentage of reduction in metabolic cost between WEC and WOEC for lifting from knee height with the Laevo HC; PWS: preferred walking speed without the exoskeleton; PWSX: preferred walking speed with the exoskeleton; RR: respiration rate; SA: standing posture, asymmetric lifting/lowering; SS: standing posture, symmetric lifting/lowering; ${\dot{VO}}_{2,norm}$: average oxygen consumption, normalized by weight; WE: with exoskeleton; WT: walking test; (^1^): value not reported, relation deduced from Figure 7 in [42]; (^2^): data not explicitly reported that should be approximately quantified from the figure; (*): significant differences from WOE.
